# Evaluating Dual Task Neurological Costs with Functional Near-Infrared Spectroscopy: A Preliminary Report in Healthy Athletes

**DOI:** 10.31083/j.jin2205133

**Published:** 2023-09-18

**Authors:** Jaclyn A Stephens, Susan Mingils, Silvia Orlandi

**Affiliations:** 1 Department of Occupational Therapy, Colorado State University, Fort Collins, CO 80524, USA; 2 Molecular Cellular Integrative Neuroscience, Colorado State University, Fort Collins, CO 80523, USA; 3 Department of Electrical, Electronic and Information Engineering ‘Guglielmo Marconi’, University of Bologna, 40136 Bologna, Italy; 4 Health Sciences and Technologies-Interdepartmental Center for Industrial Research (CIRI-SDV), University of Bologna, 40136 Bologna, Italy

**Keywords:** sports-related concussion, dual task, functional near-infrared spectroscopy, measure development

## Abstract

**Background:**

Dual task assessments, which simultaneously challenge and assess cognitive and motor performance, have been used to improve the assessment of athletes with sports-related concussions (SRC). Our lab created a Dual Task Screen (DTS) to evaluate athletes with SRCs, and we have established that it is a valid behavioral measure, as it consistently elicits poorer behavioral performance under dual, compared to single, task conditions. Here, we used a Neuroimaging-Compatible (NC) version of the DTS, named the NC-DTS, which uses portable functional near-infrared spectroscopy (fNIRS) to assess behavioral performance and neural recruitment during single and dual tasks. Our study objective was to evaluate healthy athletes and establish whether the NC-DTS is a valid dual task *neurological* assessment that can elicit different patterns of neural recruitment during dual versus single task conditions.

**Methods:**

Twenty-five healthy collegiate athletes completed the NC-DTS in a single laboratory visit. The NC-DTS includes a lower and upper extremity subtask; both include single motor, single cognitive, and dual task conditions. The NC-DTS was administered in a block design, where conditions (i.e., single motor, single cognitive, and dual task) were repeated five times to generate average behavioral performance and task-dependent neural recruitment in superficial cortical regions including: prefrontal cortex, bilateral primary motor and sensory cortices, and posterior parietal cortex. Neural recruitment was measured with fNIRS and quantified using oxygenated hemoglobin (HbO) and deoxygenated hemoglobin (HbR) metrics. A single-tailed, within subject *t*-test was used to compare average dual task behavioral performance to average single task behavioral performance. Pairwise comparisons, that were family-wise-error (FWE) corrected, were used to compare localized neural recruitment during dual versus single task conditions.

**Results:**

As observed in previous studies, the NC-DTS elicited significantly poorer behavioral performance under dual, compared to single, task conditions. Additionally, dual task conditions of the NC-DTS elicited significantly greater neural recruitment in regions of the brain associated with attention allocation and task-specific demands in three of four comparisons.

**Conclusions:**

These preliminary results suggest that the NC-DTS is a valid dual task neurological assessment which warrants future work using the NC-DTS to evaluate athletes with SRCs.

## 1. Introduction

Athletes, especially those that play contact sports, are at a heightened risk of sustaining sports-related concussions (SRC) compared to non-athletes or athletes who play non-contact sports [1]. More worrisome, however, is that once athletes are cleared to return to play after a SRC, they have a significantly increased risk of sustaining a new injury, such as a musculoskeletal injury [2] or a repeat SRC [3,4]. The risk of repeat SRCs was the focus of significant media attention in 2022, after Miami Dolphins quarterback, Tua Tagovailoa, sustained two confirmed and one suspected SRC in a single season [5]. This incident highlights the need for better return-to-play procedures for athletes of all ages and levels of play. Repeat SRCs are known to have negative long-term risks, as individuals with repeat SRCs experience symptoms for longer after sustaining a new SRC [6] and have a greater likelihood of experiencing sleep disturbances and mental health conditions than individuals without SRCs [7–10].

One way to reduce the occurrence of repeat SRCs is to delay return-to-play until an athlete is fully recovered [11]. Currently, the approach for determining return-to-play readiness after an initial SRC includes the use of subjective symptom reporting, neurological exams (including balance testing), and cognitive testing [12]. However, given that cleared athletes with recent SRCs have an increased risk for new injury [2,3], this approach appears to have insufficient sensitivity to detect ongoing deficits and may inadequately assess risk for a new injury. A potentially better approach is to use dual task assessment, which simultaneously challenges and assesses cognitive and motor performance. A body of literature has shown that dual task assessments can consistently detect residual cognitive and motor deficits in athletes with recent SRCs [13–16], suggesting enhanced sensitivity to detect vulnerabilities that may be linked to risk of future injury [17]. Despite this, it remains unclear why cleared athletes with recent SRCs have typical single task performance but significantly poorer dual task performance compared to non-concussed athletes. We hypothesize that athletes with recent SRCs may have an increased compensatory recruitment of neural attentional resources during both single and dual tasks. Subsequently, because attention, along with other related executive functions, has a limited capacity [18], the compensatory recruitment may be sufficient to support typical motor performance during single tasks, but is insufficient to sustain performance during dual tasks.

With the long-term goal of testing our compensatory recruitment hypothesis in athletes with recent SRC, our lab has focused recent efforts on developing a measure to test this hypothesis. Specifically, we developed the Dual Task Screen (DTS) [19] which can support simultaneous neuroimaging with portable functional near-infrared spectroscopy (fNIRS). Briefly, portable fNIRS systems can be used to evaluate a wide range of performance on real-world scenarios as it is less susceptible to motion artifacts than other neuroimaging methods, such as functional magnetic resonance imaging (fMRI) or electroencephalography (EEG) [20]. Through the use of near-infrared light, fNIRS detects task-dependent changes in oxygenated hemoglobin (HbO) and deoxygenated hemoglobin (HbR), which are indirect, or proxy, measures of neural activity in specific superficial cortical regions [21]. Specifically, as brain regions become more active, cerebral blood flow increases to support an increased demand for oxygen (along with other metabolites), which can be detected as decreased HbR and increased HbO [21].

Advances in fNIRS technology [22] have supported a number of recent studies where portable fNIRS systems have been paired with behavioral tasks to elicit dual task interference and evaluate neural recruitment in both younger [23,24] and older adults [25–27]. Notably, most of these studies have exclusively evaluated lower extremity motor function, whereas our DTS includes both a lower and upper extremity subtask. Each subtasks contains a single motor and a single cognitive condition, as well as a dual task condition. To date, we have conducted research with healthy athletes and established that the DTS is a valid dual task behavioral assessment, as it can elicit dual task interference (i.e., poorer behavioral performance under dual, compared to single, task conditions) in both motor [19] and cognitive [28] domains. However, we have not yet established if the Neuroimaging Compatible version of the DTS (NC-DTS) can elicit different patterns of neural recruitment during dual versus single task conditions in healthy collegiate athletes. Therefore, the primary aim of this study was to find preliminary evidence that the NC-DTS is a valid dual task neurological assessment. Notably, we defined healthy athletes as those without a recent SRC (see Materials and Methods), while recognizing that many collegiate athletes have a history of SRCs [29]. We did not expect that the NC-DTS would detect differences in athletes with and without a history of SRC. However, as a secondary aim, we sought to confirm that task-induced differences in neural recruitment were not influenced by SRC history, as we wanted the NC-DTS to be sensitive to deficits originating from recent SRC. Given previous findings in healthy younger adults [23,24], we predicted that dual task conditions on the NC-DTS would elicit greater neural recruitment in the prefrontal cortex (PFC) for both subtasks. We also anticipated that dual task conditions would elicit greater neural recruitment in task-specific regions, such as motor control or language production areas. If these predictions were confirmed in healthy athletes, this test could be used to evaluate recruitment of neural attentional resources during both single and dual tasks in athletes with recent SRC in future studies. Thus, the current study represents an essential step in addressing our long-term research objectives.

## 2. Materials and Methods

### 2.1 Study Design & Procedure

This was a cross-sectional observational study where participants attended a single laboratory visit lasting 1.5 hours where they completed a demographic questionnaire, a baseline/pre-injury version of the Immediate Post-Concussion Assessment and Cognitive Testing (ImPACT^®^; ImPACT Applications, Inc Coralville, IA, USA) test, and the Neuroimaging Compatible Dual Task Screen (NC-DTS) with simultaneous fNIRS evaluation. Only NC-DTS behavioral data and fNIRS data are included in this manuscript; thus, these measures are described in detail below. All study measures were approved by Colorado State University’s Institutional Review Board (IRB).

### 2.2 Participants

Twenty-five healthy collegiate athletes (mean age = 20.16; female: N = 16) were recruited via flyers, word-of-mouth, email listservs, and social media advertisements. The sample size was determined using a power analysis from previous behavioral data [19] with a desired effect size of 0.8, an alpha error probability of 0.05, and power of 0.95 for a one-tailed (i.e., directional) hypothesis; the sample size also mirrors that of other fNIRS and dual task studies [23,24]. Participants were considered healthy athletes if they were between the ages of 18–23, regularly engaged (at least four days/week) in organized sports, were at least six-month post any diagnosed SRC, and had no history of moderate or severe traumatic brain injury (TBI). Most participants were right-handed (N = 23). Participants played a variety of sports including: soccer (N = 5), track and field (N = 5), American football (N = 2), lacrosse (N = 2), gymnastics (N = 2), rock climbing (N = 2), rugby (N = 1), volleyball (N = 1), water polo (N = 1), baseball (N = 1), figure skating (N = 1), golf (N = 1), and rowing (N = 1). Participant history of diagnosed SRCs and/or other severe brain injuries were acquired via self-report on a demographic questionnaire. Note, the first participants recruited for this study answered a binary ‘yes/no’ question regarding SRC history. As such, the exact number and incidence dates of all past SRC are unknown for those participants, although the date of the most recent SRC was acquired. Later, the research team updated the demographic questionnaire to acquire the exact number of past SRC with incident dates. Fifteen participants had no prior SRCs (female: N = 9), and ten participants had a prior SRC (female: N = 7); three confirmed having only one prior SRC, four confirmed having two prior SRCs, but for three participants, the exact number of prior SRCs was unknown. The time since injury for participants’ most recent SRC ranged from 11 months to 8 years (mean = 2.75 years). All participants provided informed written consent.

### 2.3 Neuroimaging-Compatible Dual Task Screen (NC-DTS)

The NC-DTS was developed by our lab after the development of the original measure, the DTS. The original DTS was designed to rapidly assess dual task performance with low-cost, portable instruments [19]. We revised the measure and developed a neuroimaging-compatible version to support evaluation of the neural underpinnings of single and dual task performance [30]. The NC-DTS included a lower extremity subtask and an upper extremity subtask (LE and UE subtasks). Each subtask includes three conditions: single motor task, single cognitive task, and dual task. In the LE subtask, the single motor condition included a thirty second obstacle walk where yoga blocks were placed every five meters along a fifteen-meter walkway. During this task, the participant was instructed to walk down and back up the walkway as quickly as possible for 30 seconds, while stepping over the obstacles. The primary outcome measure was gait speed, represented as meters/second (m/s); this value was calculated by measuring the distance covered in 30 seconds and was confirmed with portable accelerometers that are strapped to the participants’ ankles. The single cognitive condition was a verbal fluency task, where participants were asked to generate as many words starting with an “easy” letter (i.e., letters ‘H, D, M, A, B, F, P, T, C, S’ for which there are a wide-range of words in English [31]) in thirty seconds; the outcome measure for this task was the number of words generated (no repeats). Finally, the dual task condition combined the obstacle walk and the verbal fluency task. The outcome measured was gait speed in m/s and unique words generated. The letters used for the verbal fluency task were counterbalanced between the single and dual tasks between participants. In the UE subtask, the single motor condition consisted of an alternating wall-toss task where participants stood 1.5 meters away from a wall and threw and caught a tennis ball from alternating hands for thirty seconds. The outcome measured was the number of successful catches. The single cognitive condition was a serial subtraction task where participants subtracted backwards by seven from a given three-digit number ending in ‘0’ or ‘5’. The outcome measured was the number of correct subtractions. Finally, the dual task condition combined the wall task and serial subtraction task, where the outcomes measured were successful catches and successful subtractions. As in the LE subtask, the numbers used for the serial subtrack task were counterbalanced between the single and dual tasks between participants. To support evaluation of neural activation during the single and dual tasks, the three conditions (i.e., single motor, single cognitive, and dual task) were repeated five times, for a total of fifteen trials in a randomized block design during fNIRS acquisition. The stimulus presentation software PsychoPy, version 3 (Open Science Tools Ltd., Nottingham, Nottinghamshire, UK) [32] was used to randomize trials and display trial order to a member of the research team, who then instructed participants on how and when to begin trials. PsychoPy also sent trial marker information to the fNIRS acquisition software (see details below) via a lab streaming layer. All behavioral performance was video recorded and scored by two trained members of the research team. Detailed scoring procedures are outlined in a previous publication [30].

### 2.4 Statistical Analysis of Behavioral Data

To confirm that, like prior versions of the DTS, the NC-DTS could elicit dual task interference, behavioral data were analyzed. Specifically, behavioral performance on the NC-DTS was evaluated by first averaging the performance on the five trials for each of the three conditions in the LE and UE subtask. Specifically, for the LE subtask, we used average gait speed as the single motor condition performance metric and average number of words for the single cognitive condition performance metric; average gait speed and average number of words were the dual task condition performance metrics. Similarly, for the UE subtask, we used average number of catches as the single motor condition performance metric and average number of subtractions as the single cognitive condition performance metric; average number of catches and average number of subtractions were the dual task condition performance metrics. For both subtasks, we tested for dual task motor and cognitive interference using a single-tailed, paired *t*-test that compared average dual task condition performance to average single task condition performance. A single-tailed *t*-test was selected as we hypothesized, *a priori*, that dual task performance would be poorer than single task performance. As two *t*-tests were completed for each subtask, we set our alpha level at a corrected value of *p* = 0.025. Finally, we used independent *t*-tests to test for potential differences in dual task performance [33] (see calculation below) between athletes with a history of SRC (N = 10) and athletes without a history of SRC (N = 15). Note, dual task performance—rather than dual or single task condition performance—is a better metric for between-subject analyses as it accounts for within-subject variability on single task performance.


$$\text{Dual}\;\text{Task}\;\text{Effect}=\frac{\left(\text{Dual}\;\text{Task}\;\text{Performance}-\text{Single}\;\text{Task}\;\text{Performance}\right)}{\text{Single}\;\text{Task}\;\text{Performance}}$$

### 2.5 Functional Near-Infrared Spectroscopy (fNIRS) Acquisition

**Fig. 1.**
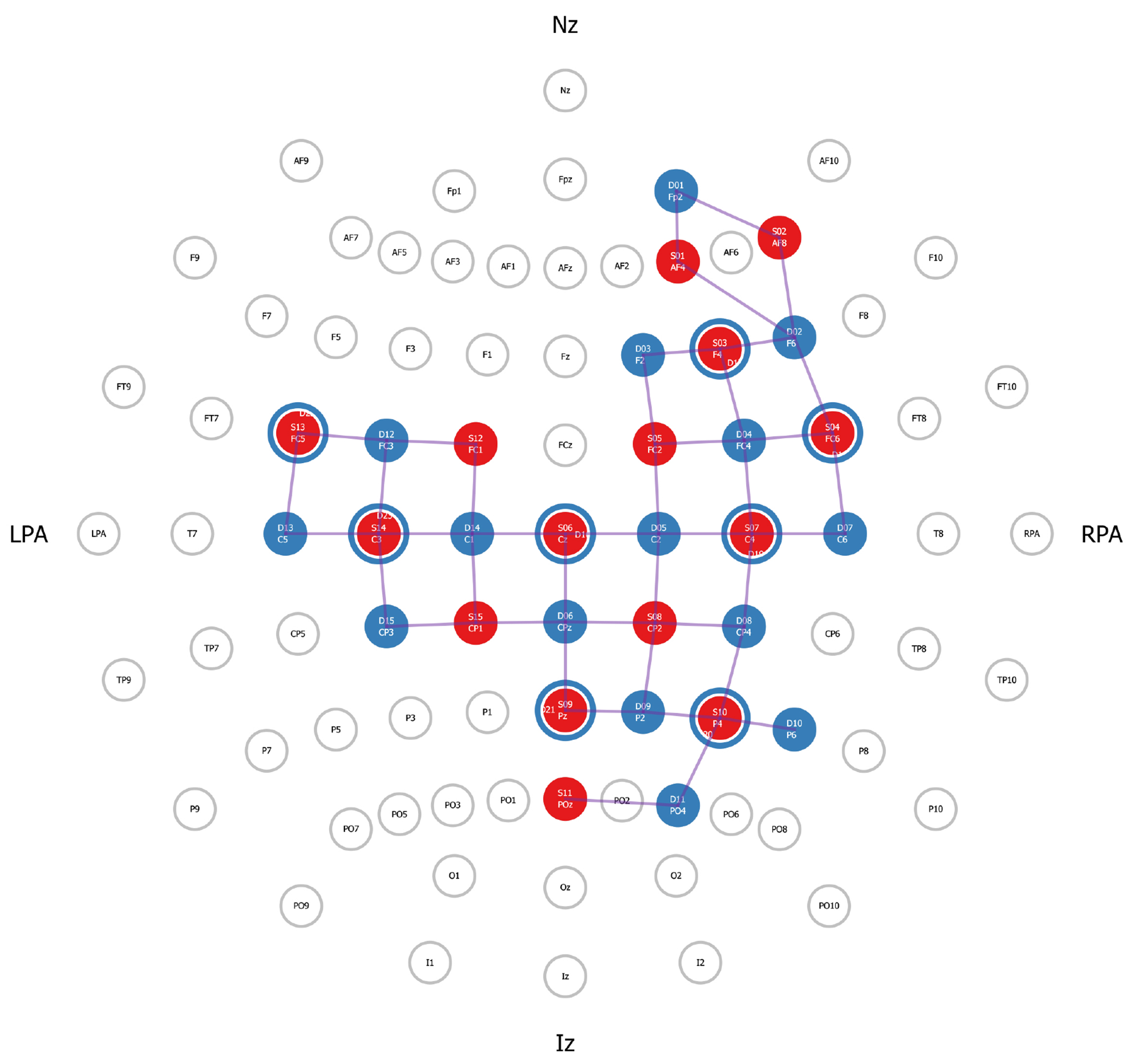
Functional near-infrared spectroscopy (FNIRS) head probe. The use of a portable fNIRS device, which has limited head coverage, required that regions of interest (ROIs) be established *a priori*. For this study, 15 sources and 15 detectors were used to create 42 channels situated over right lateralized nodes of the frontoparietal attention network and over bilateral motor and sensory regions. Additionally, 8 short-separator channels (depicted as blue rings around red circles) were placed throughout the head probe to measure scalp perfusion. Anatomical reference points are: nasion (Nz), right pre-auricular (RPA), inion (Iz) and left pre-auricular (LPA).

fNIRS data were acquired with a NIRSport2 (NIRx Medical Technologies, Berlin, German, https://nirx.net/), which is a wearable device that is secured to participants’ backs with backpack-like straps and buckles. As the NIR-Sport2 is a portable device, it does not provide full head coverage. Rather, the head probe was designed to measure regions of interest (ROI), established *a priori*. To design our montage, we used the AAL2 atlas within the fNIRS Optodes Location Decider (fOLD) toolbox [34] in Matlab to identify channel locations above superficial components of the right lateralized frontoparietal attention network [35] along with sensory and motor regions. In total, the fNIRS head probe included 30 optodes, 15 LED-sources (760 and 850 nanometers) and 15 detectors, which created 42 channels over the ROIs. Additionally, eight short-separator detectors were placed on the interior of the cap to create an additional eight channels to measure scalp perfusion [36]. The anatomical landmarks for each channel were confirmed using AtlasViewer (Neurophotonics, Boston, MA, USA) [37]; see Fig. 1 and Table 1. All fNIRS data were acquired and wirelessly transmitted to a laptop using Aurora Version 2021.9 (NIRx Medical Technologies, Berlin, Germany, https://nirx.net/software) software. Prior to data acquisition, a signal optimization step was completed to calibrate the amount of light needed for each light source and assess if high-quality data could be acquired at each primary short-separator channel. The Aurora interface displayed light source intensities for each source and indicators of ‘critical’, ‘acceptable’, or ‘excellent’ quality at each channel. These indicators reflect how much light is passing through tissues measured in millivolts (mV). An ‘excellent’ quality indicator reflects values greater than 3 mV; ‘acceptable’ reflects values between 0.5 mV and 3 mV, and critical values (when data should not be acquired) were below 0.5 mV. Signal quality was further evaluated using a coefficient of variance, calculated as the ratio between the standard deviation of the raw signal, measured over 1.5 seconds of data. Excellent coefficient of variance values were less than 2.5%; acceptable values were between 2.5% and 7.4%, and critical values (when data should not be acquired) were at or above 7.5%. For all participants, data acquisition did not start until all channels, including short separator channels, reached acceptable or excellent levels for both quality indicators. Data were acquired at a 4.65 Hz sampling rate.

### 2.6 fNIRS Data Pre-Processing and Data Extraction

Raw fNIRS data were wirelessly transmitted to a designated computer and processed using a novel commercial software tool, Satori Version 1.8 by Brain Innovation (NIRx Medical Technologies, https://nirx.net/satori). Satori is a user-friendly fNIRS analysis software that includes a Graphical User Interface (GUI), which supports easy selection of pre-processing steps such as motion artifact removal, physiological noise detection, and channel selection and removal. After uploading the .nirs file containing raw data in Satori, preprocessing steps of conversion and spatial registration were automatically performed by the software. Specifically, the raw light intensity data were converted to optical density values and then converted to oxygenated hemoglobin (HbO), deoxygenated hemoglobin (HbR), and total hemoglobin (HbT) values using Modified Beer-Lambert Law [38]. All data were spatially registered to our head probe (see Fig. 1) and displayed for visual inspection. Following these automated steps, we updated event markers files for each subject by renaming the event marker data that was generated by PsychoPy. Specifically, during fNIRS acquisition, PsychoPy sent numerical trial markers that corresponded with trials names to Aurora via a lab streaming layer. Therefore, in this step, we changed the numerical trial markers to the corresponding event name (e.g., “1” indicated “Single Motor” in the LE subtask) and indicated that all trials had a duration of 30 seconds. The event marker file was then saved and applied to the data. Next, temporal pre-processing steps were completed using a GUI. Motion artifact detection and correction were applied with a spike removal procedure using the Satori default parameters (10 interactions, 5 s lag, 3.5 threshold, 0.5 influence). In the instance of spike detection, a monotonic interpolation was applied. The Temporal Derivative Distribution Repair (TDDR) [39] was then applied to restore high frequency bands.

Physiological noise removal was completed in three steps. First, short separation regression (SSR) was completed through a generalized linear model (GLM), which uses the highest correlation method to automatically select channels with artifacts. Next, temporal filtering was completed to remove low-frequency drifts as well as part of the non-hemodynamic related signal components, such as heart rate, using a Butterworth high-pass filter followed by a Gaussian low-pass smoothing filter with cut-off frequencies of 0.01 Hz and 0.4 Hz, respectively.

Finally, to make our data comparable, we applied a normalization step using the Z-Transform. Due to the lack of previous studies conducted with Satori, we did not use the default setting that automatically rejects channels with a scalp coupling index (SCI) below 0.75 [40], which indicates that data originating from the light sources was poorly correlated. Instead, we processed all data without automated channel rejection (these files were used for group-level analysis) before processing raw data with the channel rejection step. In doing so, Satori generated a channel rejection map that indicated which channels would have been rejected and their SCI value. We used this information to check our data for potential outliers after group-level analysis (see fNIRS data analysis section). After all preprocessing was completed, a multi-subject GLM approach was applied within Satori to generate group-level data.

### 2.7 fNIRS Statistical Analysis

Separate output files were generated by Satori for the LE and UE subtasks. Each subtask file included average HbO, HbR, and HbT (not analyzed) beta values for each participant, at each channel, and for each condition (i.e., dual, single motor, and single cognitive). These data were exported into SPSS Statistics Version 26 (IBM Corp., Chicago, IL, USA) for analyses. To test for potential differences in HbO or HBR during dual versus single motor conditions, two repeated-measures ANOVAs* (one for HbO and one for HbR) were used to conduct pairwise comparisons at all 42 channels; these were Sidak corrected to reduce familywise error (FWE) and account for multiple comparisons [41]. The adjusted alpha level was set at *p* < 0.05. Similarly, to test for potential differences in HbO or HBR during dual versus single cognitive conditions, two repeated-measures ANOVAs (one for HbO and one for HbR) were used to conduct pairwise comparisons at all 42 channels; these were also Sidak corrected to account for multiple comparisons, and the adjusted alpha level was set at *p* < 0.05. As the primary control analysis, in instances where significant differences were observed between channels, participants with SCI values <0.75 were removed from analysis to confirm that they were not unduly influencing results for each significant channel. Additionally, as with behavioral data, a between subjects factor of group (uncorrected for multiple comparisons) was added to the repeated measures ANOVA to test for potential differences in athletes with and without a history of SRC to address our secondary aim.

*Note: the pairwise comparisons generated by the ANOVA are analogous to multiple within-subjects *t*-tests that we used to compare dual and single task HbO/HbR values at each channel. However, SPSS software does not generate Sidak adjusted *p*-values with a multiple within-subject *t*-test approach; thus, the repeated measures ANOVA was used to acquire the desired outcomes.

## 3. Results

### 3.1 Behavioral Results on LE and UE Subtasks

**Fig. 2.**
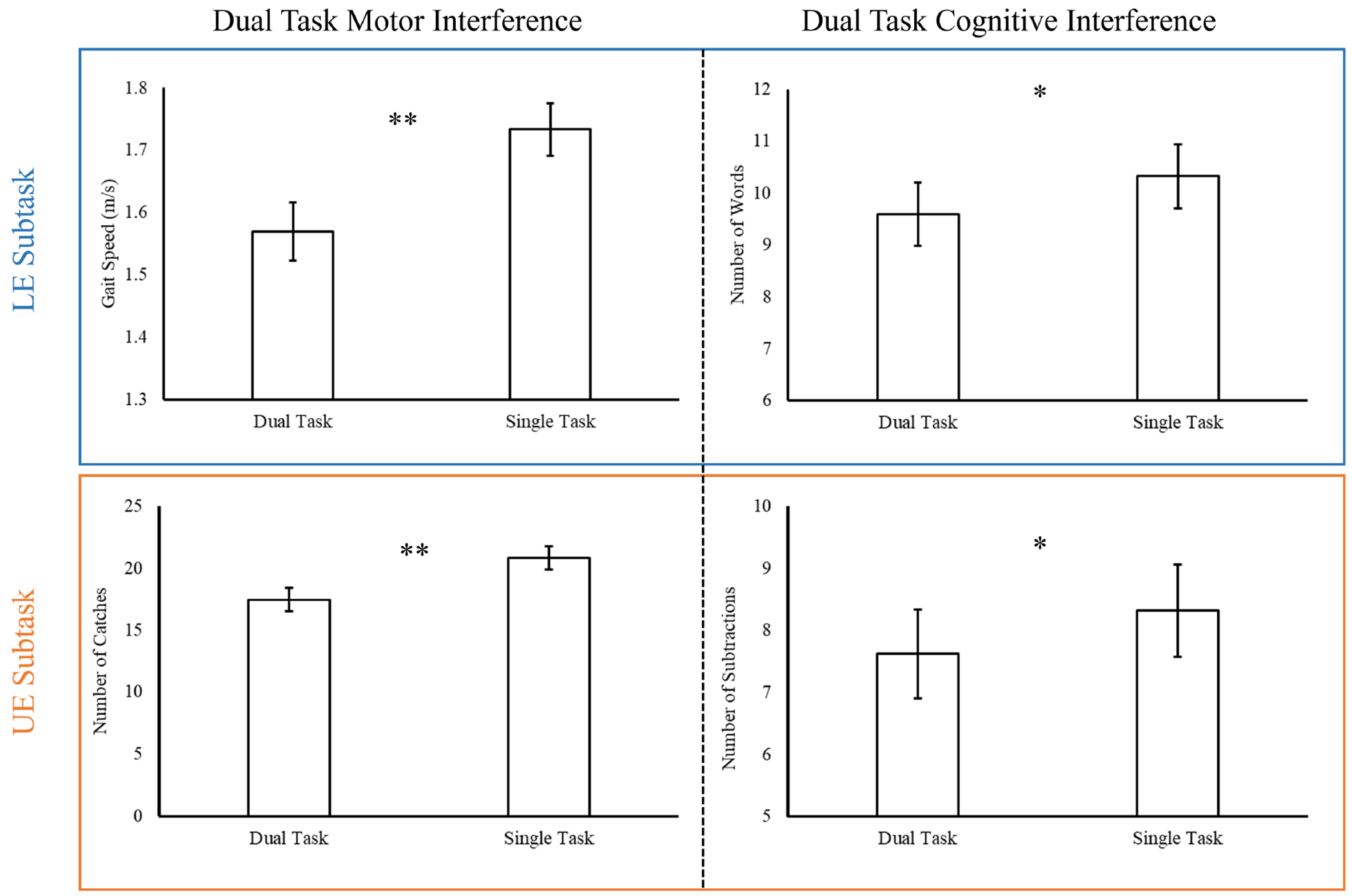
Behavioral results on the LE and UE subtasks. Dual task motor interference was observed on both the LE and UE subtasks, as motor performance was significantly poorer during dual tasks compared to single tasks. Likewise, dual task cognitive interference was observed on both the LE and UE subtasks, as cognitive performance was significantly poorer during dual tasks compared to single tasks. * indicates *p* < 0.01, ** indicates *p* < 0.001. LE, lower extremity; UE, upper extremity.

Consistent with previous findings [19], dual task interference was observed on the LE subtask; participants had significantly slower gait speed during dual task conditions (mean = 1.57 m/s, standard error (SE) = 0.05) compared to single task conditions (mean = 1.73 m/s, SE = 0.04; *p* < 0.001) and generated significantly fewer words during dual task conditions (mean = 9.59, SE = 0.61) compared to single task conditions (mean = 10.33, SE = 0.62; *p* = 0.006). Similarly, on the UE subtask participants had significantly fewer catches during dual task conditions (mean = 17.44, SE = 0.97) compared to single task conditions (mean = 20.84, SE = 0.95; *p* < 0.001) and had significantly fewer subtractions during dual task conditions (mean = 7.61, SE = 0.71) compared to single task conditions (mean = 8.32, SE = 0.75; *p* = 0.005); see Fig. 2. Results from between-group analyses showed no significant difference between groups on the LE subtask for dual task motor performance (*p* = 0.282) or dual task cognitive performance (*p* = 0.944). Likewise, there were no significant differences on the UE subtask between athletes with a history of SRC and athletes without a history of SRC on dual task motor performance (*p* = 0.292) or on dual task cognitive performance (*p* = 0.682).

### 3.2 fNIRS Results from LE Subtask

**Fig. 3.**
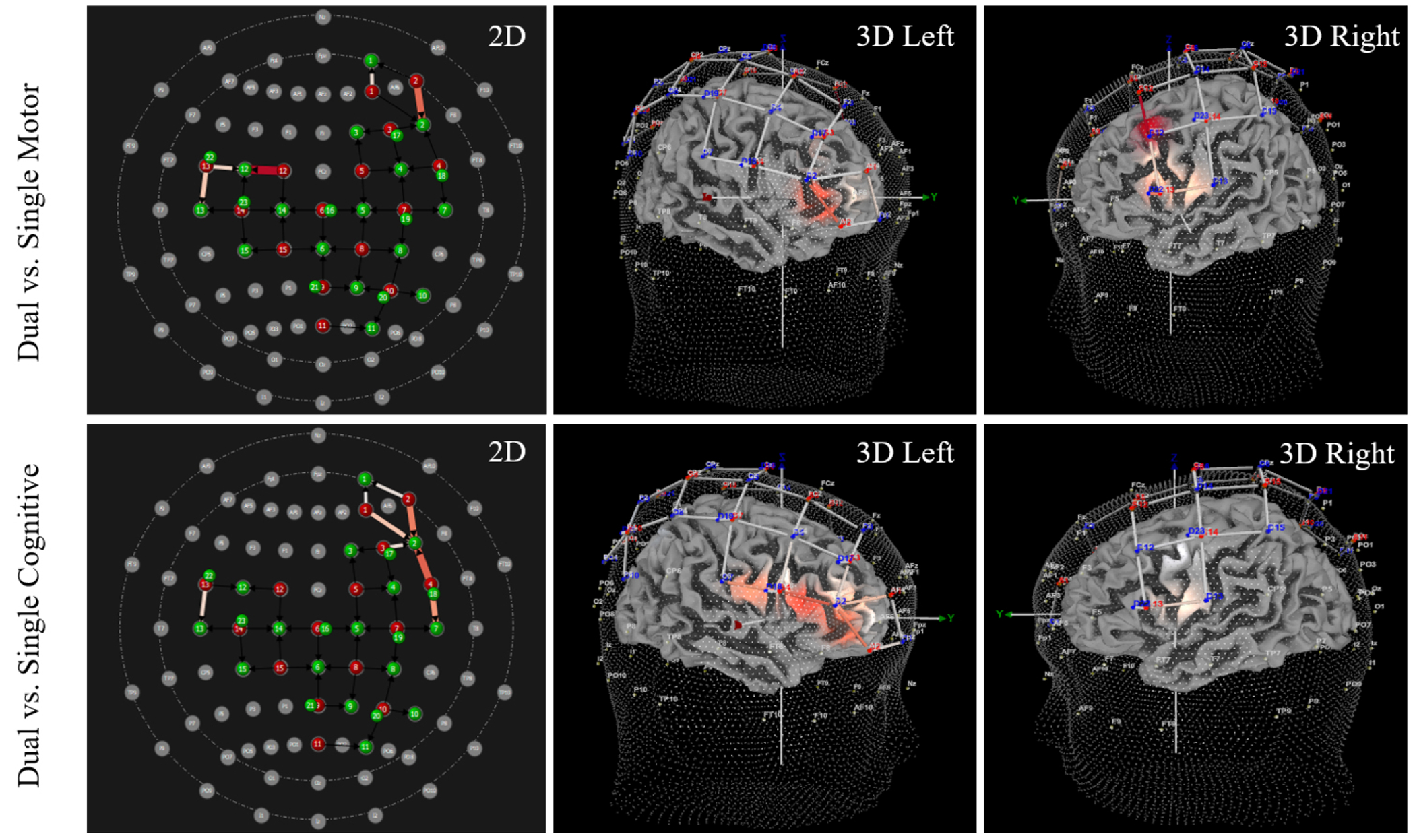
Lower extremity subtask elicits significantly increased oxygenated hemoglobin (HbO) during dual tasks. Significantly increased HbO was observed during dual tasks compared to single motor and single cognitive tasks. The 2D and 3D images above illustrate the locations where increased HbO was observed, and the light intensity indicates the magnitude of difference. Specifically, deeper shades of red indicates a greater magnitude in the difference between dual and single tasks, whereas lighter shades represent smaller, but significant, differences. See Table 1 for location and beta values of each channel.

Pairwise comparisons identified channels with significant differences in HbO. Significantly increased HbO was observed during the dual task condition compared to the single motor condition at channels 1, 4, 38, 40, and 41. The precise regions with increased HbO (as per the AAL atlas [42]) included: right dorsolateral superior frontal gyrus, right inferior frontal gyrus (triangular part), left middle frontal gyrus, and left inferior frontal gyrus (opercular part). Additionally, significantly increased HbO was observed during dual task conditions compared to single cognitive conditions at channels 1, 2, 3, 4, 5, 9, 11, and 41. The precise regions included: right dorsolateral superior frontal gyrus, right middle frontal gyrus, right inferior frontal gyrus (triangular part), right precentral gyrus, and left inferior frontal gyrus (opercular part). See Table 2 for HbO values, results, and SCI control analysis outcomes. Fig. 3 provides a visualization of these findings. Between-group analyses indicated no significant difference between athletes with and without a history of SRC at any channel (all *p*-values > 0.083, range 0.083–0.930). The multivariate results also indicated no significant group x task interaction (F _(1,2)_ = 0.956, *p* = 0.584).

**Fig. 4.**
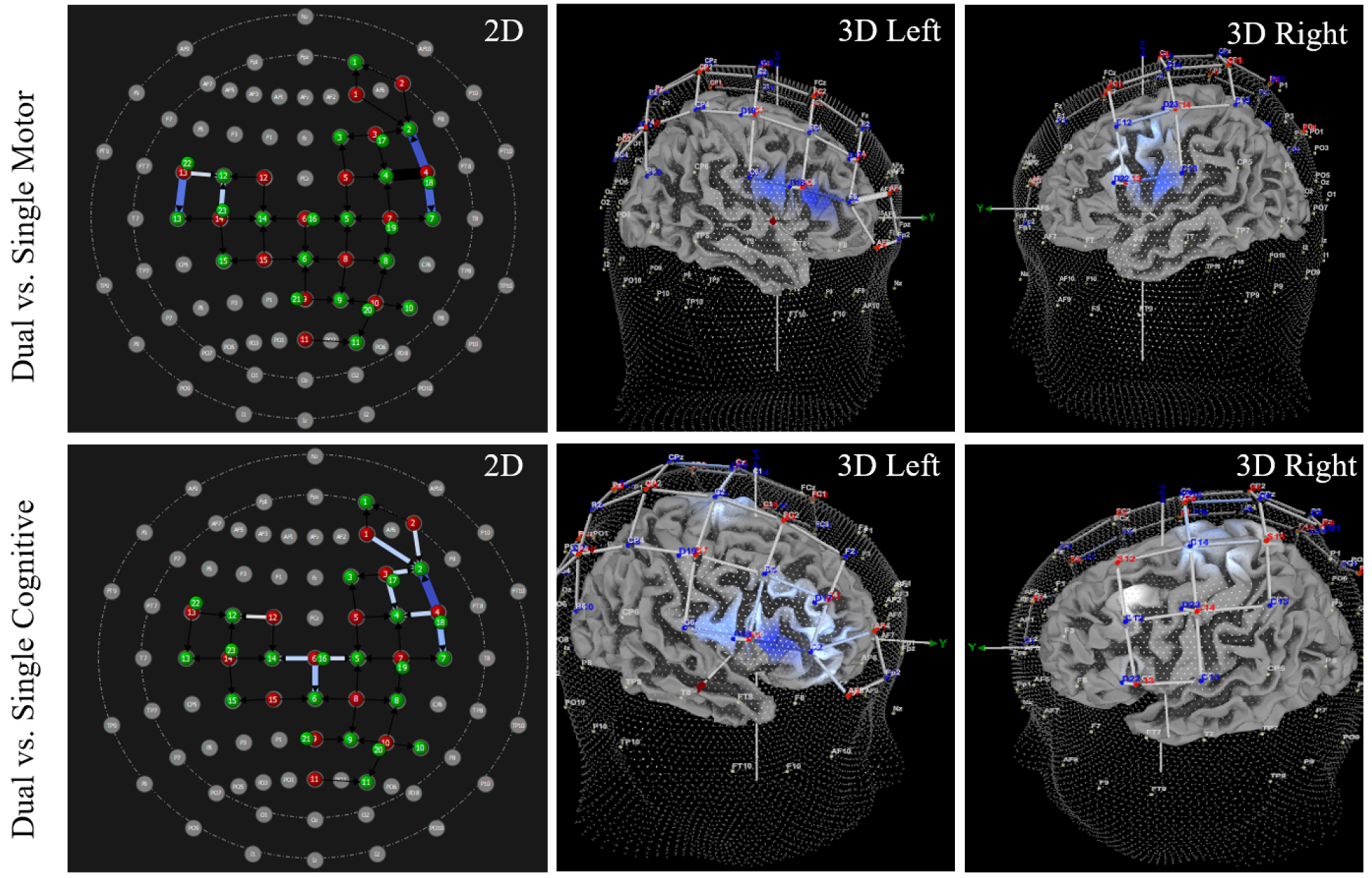
Lower extremity subtask elicits significantly decreased deoxygenated hemoglobin (HbR) during dual tasks. Significantly decreased HbR was observed during dual tasks compared to single motor and single cognitive tasks. The 2D and 3D images above illustrate the locations where decreased HbR was observed, and the light intensity indicates the magnitude of difference. Specifically, deeper shades of blue indicates a greater magnitude in the difference between dual and single tasks, whereas lighter shades represent smaller, but significant, differences. See Table 2 for location and beta values of each channel.

Results from pairwise comparisons identified channels with significant differences in HbR. Significantly decreased HbR was observed during the dual task condition compared to the single motor condition at channels 9, 11, 40, 41, and 43. The precise regions with decreased HbR included: right inferior frontal gyrus (triangular part), left and right precentral gyri, and left inferior frontal gyrus (opercular part). Additionally, significantly decreased HbR was observed during dual task conditions compared to single cognitive conditions at channels 2, 4, 5, 7, 9, 11, 17, 18, and 38. The precise regions included: right middle frontal gyrus, right inferior frontal gyrus (triangular part), right middle frontal gyrus, right precentral gyrus, left middle frontal gyrus, and left paracentral lobule. See Table 3 for HbR values, results, and SCI control analysis outcomes, and see Fig. 4 for visualization of findings. Between-group analyses indicated no significant difference between athletes with and without a history of SRC at any channel (all *p*-values > 0.097, range 0.097–0.984). The multivariate results also indicated no significant group x task interaction (F _(1,2)_ = 0.908, *p* = 0.630).

### 3.3 fNIRS Results from UE Subtask

**Fig. 5.**
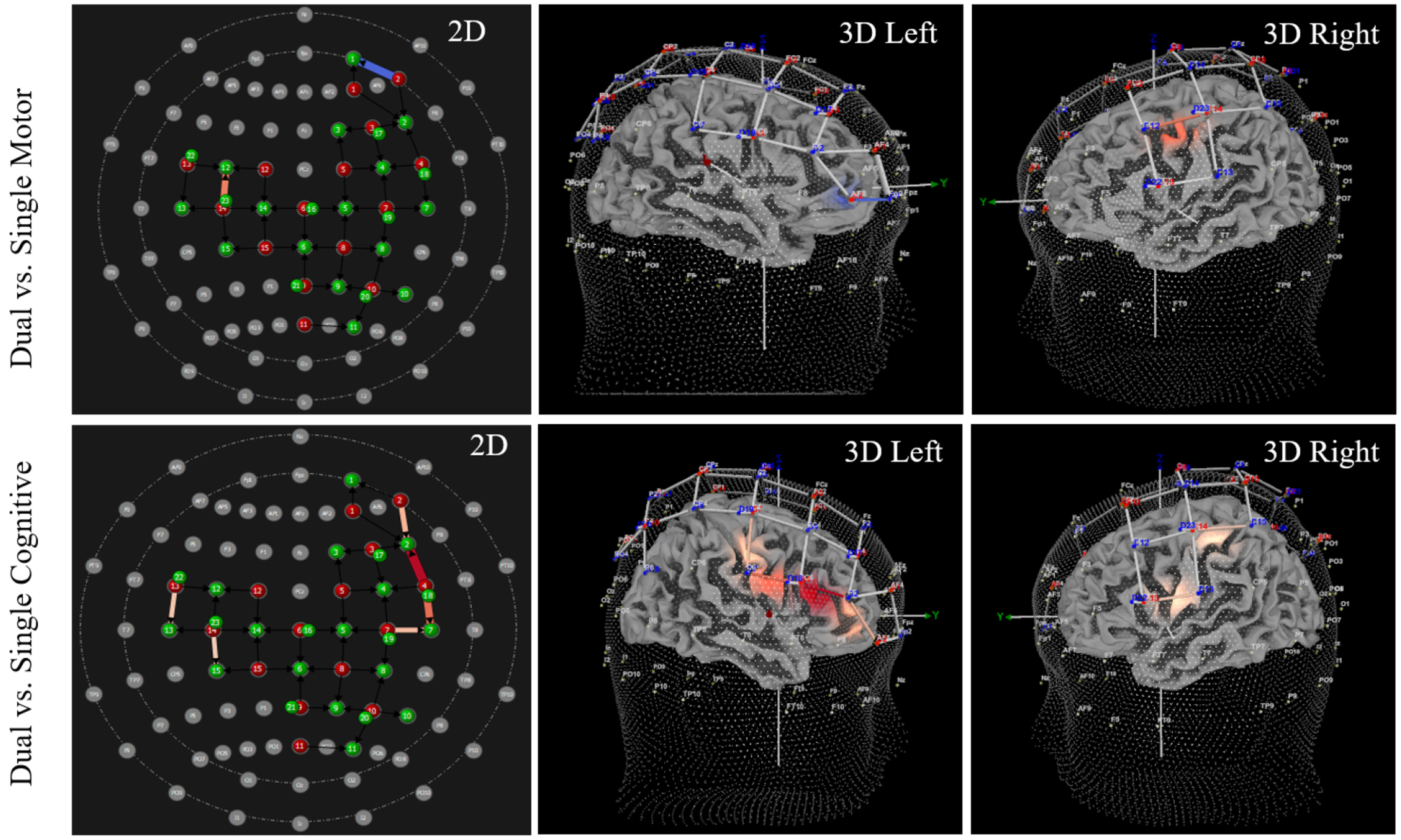
Upper extremity subtask elicits inconsistent HbO patterns during dual tasks. On the UE subtask, the dual task condition, compared to the single motor condition, elicited increased HbO in one channel (shown in red) but significantly decreased HbO in another channel (shown in blue). However, the dual task condition, compared to the single cognitive condition, consistently elicited significantly greater HbO. The 2D and 3D images above illustrate the locations where increased and decreased HbO were observed, and the light intensity indicates the magnitude of difference. Specifically, deeper shades of red (or blue) indicates a greater magnitude in the difference between dual and single tasks, whereas lighter shades represent smaller, but significant, differences. See Table 3 for location and beta values of each channel.

Results from pairwise comparisons identified channels with significant differences in HbO. Significantly increased HbO was observed during the dual task condition compared to the single motor condition at channel 43 (specifically the left precentral gyrus), and significantly decreased HbO was observed during the dual task condition at channel 3 (specifically, right middle frontal gyrus). Additionally, significantly increased HbO was observed during dual task conditions compared to single cognitive conditions at channels 4, 9, 11, 22, 41, and 46. Precisely, these regions included: right inferior frontal gyrus (triangular part), right precentral gyrus, and left inferior frontal gyrus (opercular part); significant findings in right postcentral gyrus and left inferior parietal gyrus were negated with control analyses. See Table 4 for HbO values, results, and SCI control analysis outcomes, and see Fig. 5 for visualization of findings. Between-group analyses indicated no significant difference between athletes with and without a history of SRC in 41 of 42 channels (*p*-values > 0.077, range 0.077–0.987). In channel 39, there was a significant difference detected (*p* = 0.038). However, this *p*-value had not been adjusted for multiple comparisons, and the multivariate results indicated no significant group x task interaction (F (_1,2_) = 1.317, *p* = 0.310), so this between-group difference was not further evaluated.

**Fig. 6.**
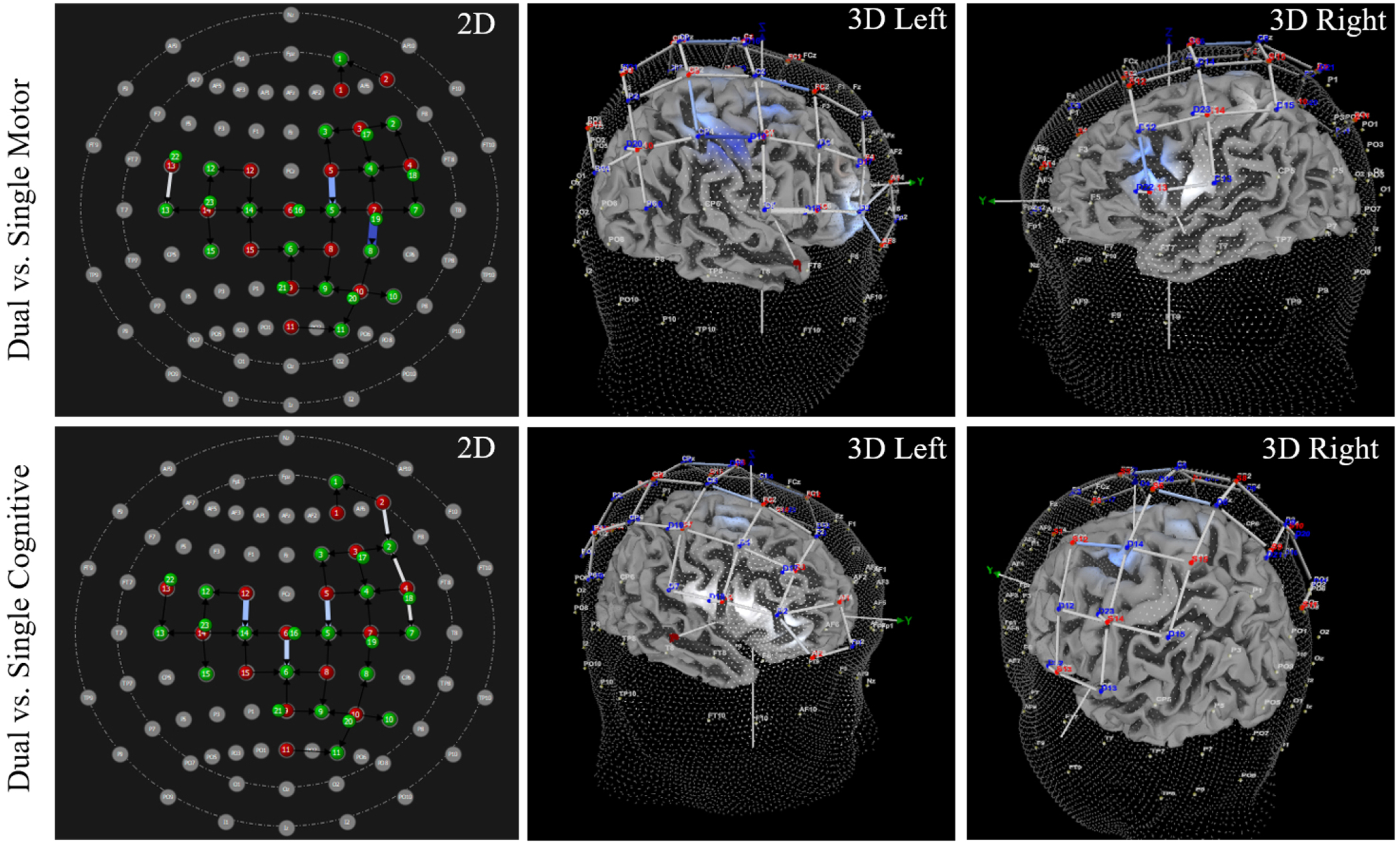
Upper extremity subtask elicits significantly decreased HbR during dual tasks. Significantly decreased HbR was observed during dual tasks compared to single motor and single cognitive tasks. The 2D and 3D images above illustrate the locations where decreased HbR was observed, and the light intensity indicates the magnitude of difference. Specifically, deeper shades of blue indicates a greater magnitude in the difference between dual and single tasks, whereas lighter shades represent smaller, but significant, differences. See Table 4 for location and beta values of each channel.

Results from pairwise comparisons identified channels with significant differences in HbR. Significantly decreased HbR was observed during the dual task condition compared to the single motor condition at channels 15, 23, and 41. The specific regions with decreased HbR included: right dorsolateral superior frontal gyrus, left inferior frontal gyrus (opercular part), and right postcentral gyrus, although the difference in right postcentral gyrus was negated after conducting control analyses. Additionally, significantly decreased HbR was observed during dual task conditions compared to single cognitive conditions at channels 4, 9, 11, 15, 17, and 39. Precisely, these regions included: right and left dorsolateral superior frontal gyrus, right inferior frontal gyrus (triangular part), right precentral gyrus, and left paracentral lobule. See Table 5 for HbR values, results, and SCI control analysis outcomes, and see Fig. 6 for visualization of findings. Between group analyses indicated no significant difference between athletes with and without a history of SRC in 40 of 42 channels (*p* values > 0.063, range 0.063–0.990). There were significant differences detected between athletes with and without a history of SRC in channel 3 (*p* = 0.045) and in channel 29 (*p* = 0.049). However, these *p*-values had not been adjusted for multiple comparisons, and a multivariate results indicated no significant group x task interaction (F(_1,2_) = 0.668, *p* = 0.859). Therefore, these between-group differences were not further evaluated.

Note for Figs. 3,4,5,6: All 3D images were angled to optimize viewing of channels with significant increases/decreases in HbO or HbR. As such, there are intentional inconsistencies in how 3D images appear within and between figures.

## 4. Discussion

The primary aim of this study was to find preliminary evidence that the NC-DTS is a valid dual task neurological assessment. We operationally defined this as: an assessment that elicits different patterns of neural recruitment during dual versus single task conditions. This mirrors our previous work with the DTS, where we confirmed that the DTS was a valid dual task behavioral assessment by showing it could elicit dual task motor [19] and cognitive [28] interference, or poorer behavioral performance during dual versus single task conditions. Notably, the NC-DTS also elicited dual task interference on both subtasks (see Fig. 2), despite modifications that supported simultaneously neuroimaging. More importantly, however, the NC-DTS elicited significantly different patterns of neural recruitment during dual versus single task conditions, which supports that it is a valid dual task neurological assessment in healthy athletes. Further, many observed differences in neural recruitment were in alignment with our predictions; these are outlined below. The secondary aim of the study was also successfully accomplished revealing no significant distinctions between athletes with and without a prior history of SRC. These outcomes suggest that task-induced differences in neural recruitment, as assessed by the NC-DTS, were not significantly impacted by athletes’ SRC history in this particular sample. These findings hold great importance for forthcoming studies involving athletes with recent SRCs, as any observed differences in neural recruitment could be more accurately attributed to their recent SRC rather than their SRC history.

In the LE subtask, we found that the dual task condition elicited significantly greater increases in HbO in right and left PFC, compared to the single motor condition. Likewise, we observed significantly greater decreases in HbR in right and left PFC and bilateral primary motor cortices. It is not surprising that there is substantial overlap in regions where increased HbO and decreased HbR were observed, as greater neural recruitment is reflected by decreases in HbR and subsequent increases in HbO [21]. These results align with our predictions that dual task conditions would elicit greater recruitment of PFC, which has been observed in other studies with healthy adults [23,24]. This is a logical and expected finding as one of the roles of the PFC is to control attention allocation given task demands [43]. Additionally, given the distinct task demands of the NC-DTS, we expected to see greater recruitment in regions associated with language, as a verbal fluency task was added to the obstacle walk to create the dual task condition. As expected, we observed significantly greater recruitment of left inferior frontal gyrus, which is a key region for language production [44]. When we compared the dual task condition to the single cognitive condition, we observed significantly greater increases in HbO in right PFC, left PFC, and right motor cortex. Likewise, we observed significantly greater decreases in HbR in right and left PFC, right motor cortex, and left paracentral lobule. As with single versus dual motor performance, we expected and observed greater recruitment of PFC, consistent with previous findings [23,24]. Additionally, given task demands, we expected to see greater recruitment in regions associated with motor control, as an obstacle walk was added to the verbal fluency task to create the dual task condition. Indeed, we observed increased recruitment of the right motor cortex, which is associated with left lateralized motor control [45], and the left paracentral lobule, which is associated with right lower limb motor control [46].

In the UE subtask, when comparing the dual task condition to the single motor condition, we did not observe expected patterns of neural recruitment. Our predictions were exploratory as, to our knowledge, we are one of the only labs conducting dual task paradigms with upper extremity motor tasks. Unlike the dual task of the LE subtask, the dual task of the UE subtask, as compared to the single motor task, only elicited increased HbO in left motor cortex, but this finding was negated after conducting control analyses. We also found decreased HbO in the right PFC, which contradicted our predictions. However, we did observe significant decreases in HbR in the right and left PFC, which is reflective of increased neural activity. As in the LE subtask, we had anticipated seeing increased recruitment of the PFC, which was partially supported by HbR results, but not the HbO results. We also predicted increased recruitment to align with task demands; namely we would have anticipated seeing greater recruitment of regions associated with serial subtraction, such as the right inferior frontal gyrus, angular gyri, and supramarginal gyri [47], but this was not observed. Our lack of task-specific findings could be contributed to the limited head coverage of our portable fNIRS system. For example, our head probe was not positioned to detect recruitment of left or right supramarginal gyri, nor the left angular gyrus (see Fig. 1). It is possible that a head probe with greater coverage would be able to detect increased recruitment of regions associated with serial subtraction. However, our lack of robust findings in the PFC cannot be attributed to the head probe and will require further exploration in future work.

Despite this, when contrasting dual task to single cognitive conditions, we did observe significantly greater increases in HbO in the right and left PFC, and the right primary motor cortex. Similarly, we observed significantly decreased HbR in the right and left PFC, the right primary motor cortex, and the left paracentral lobule. These findings were aligned with our predictions of recruitment of the PFC during a dual task. Additionally, given task demands, we expected to see greater recruitment of regions associated with motor control [45]. This was partially supported, as greater HbO and decreased HbR were observed in the right, but not left, primary motor cortex. Instead, significantly decreased HbR was found in the left paracentral lobule, which was unexpected as this region is primarily associated with lower extremity limb control [46]. Notably, the wall-toss task required participants to catch and throw with both hands, and the majority (92%) of our participants were right-handed. Perhaps, the heightened recruitment of the right primary motor cortex reflected increased effort to execute the ball toss and catch with the left hand. Future studies should explore if left-handed individuals recruit left-lateralized motor regions to execute this task. It should also be noted that the wall-toss task requires hand-eye coordination, which is a skill primarily supported by the cerebellum [48]. However, the cerebellum was not covered by our head probe.

## 5. Limitations & Future Directions

Within our summary and interpretation of findings, we noted a few instances where predicted outcomes were not observed and unexpected outcomes were found. Given the relative novelty of our approach, this is not entirely surprising, but certainly warrants future work. As a logical first step, futures studies should replicate and confirm that the NC-DTS elicits similar patterns of neural recruitment during dual versus single tasks in a unique sample of participants. Additionally, we may consider refining our head probe to better detect neural recruitment in additional regions, such as the left parietal lobe or cerebellum. However, this would require us to move optodes from established regions to new regions, as our current device cannot support expanded head coverage. This is a known limitation of portable fNIRS systems [20], but with continued advances in the field, we anticipate our future studies could incorporate an fNIRS device with expanded head coverage that would allow us to refine our head probe by adding, rather than moving, optodes.

The sample of participants used for this study may have introduced additional limitations. For example, our method for evaluating SRC history was suboptimal. At the beginning of the study, we asked participants a binary ‘yes/no’ regarding their SRC history and only later began inquiring about the exact number of past SRCs and their incidence dates. As such, at least three of ten participants with a history SRC had only one injury, and for another three participants, the exact number of prior SRCs is unknown. Thus, we may have been underpowered or less likely to detect deficits originating from multiple SRCs. Additionally, we included athletes from a wide range of sports, including non-contact sports where concussion incidence is lower and sub-concussive impacts are rare [1]. Thus, participant heterogeneity could have reduced our ability to detect if SRC history did indeed influence task-based differences in neural recruitment. In our future work, collecting a complete history from participants and potentially including a more homogenous sample will improve our capacity to measure potential differences between athletes with no prior SRCs and those with multiple SRCs.

A final potential limitation of our study is use of a new proprietary analysis software Satori (NIRx.net). Although there are published recommendations for best practice [49], the relative newness of fNIRS studies, particularly with portable systems, has resulted in the absence of a ‘gold standard’ for data reduction and processing. Satori allows researchers to upload and process data using GUIs that do not require extensive computer programming skills. This software has been tested for fNIRS data processing and statistical analysis [50], and incorporates a number of processing options that have been empirically validated. However, in previously published work, fNIRS data reduction and analyses have been conducted using proprietary software from other fNIRS device companies, such as OxySoft (https://www.artinis.com) or via Matlab toolboxes, such as Homer, Homer2, Homer3 [51], Statistical Parametric Mapping (SPM) for fNIRS [52], fNIRS NIRSTORM based on MEEG Brainstorm [53], BrainAnalyzIR [34] and Python toolboxes, such as MNE/NIRS for fNIRS data [54,55]. In general, these toolboxes require more computer programming expertise and may require users to complete analysis steps across different programs. However, researchers are virtually unlimited in their selection of processing steps and can select parameters that are optimal for their data. In contrast, Satori has a more limited selection of processing parameters, and end-users are reliant on developers to incorporate new processing parameters and options. Nonetheless, use of programs like Satori could support increased consistency in fNIRS data analysis, allowing researchers across labs to compare findings more readily and improve generalizability of findings. As fNIRS research continues to expand, it may become more apparent which approach, either the use of toolboxes and customized processing pipelines or use of proprietary software programs with more standardized processing pipelines, is optimal for analyzing and reproducing fNIRS findings.

## 6. Conclusions

Here, we observed that the dual task conditions of the NC-DTS elicited significantly greater neural recruitment in regions of the brain associated with attention allocation [43] and task-specific demands in three out of four comparisons. These encouraging findings provide preliminary evidence that the NC-DTS is a valid dual task for neurological assessment. Subsequently, future studies should be completed using the NC-DTS to determine if athletes with recent SRC have increased compensatory recruitment of neural attentional resources during complex task performance and if these patterns of compensatory recruitment are associated with subsequent injury risk.

## Figures and Tables

**Table 1.** FNIRS head probe—location of channels, sources, and detectors.

Channel	Source	Detector	Channel coordinates (MNI)	AAL Location
1	1	1	25 58 10	Frontal_Sup_R
2	1	2	37 50 14	Frontal_Mid_R
3	2	1	34 63 0	Frontal_Mid_R
4	2	2	44 46 2	Frontal_Inf_Tri_R
5	3	2	42 34 19	Frontal_Mid_R
6	3	3	32 47 43	Frontal_Mid_R
7	3	4	41 20 30	Frontal_Inf_Tri_R
8	3	17	34 30 27	Frontal_Mid_R
9	4	2	46 24 19	Frontal_Inf_Tri_R
10	4	4	63 20 36	Frontal_Inf_Oper_R
11	4	7	65 6 23	Precentral_R
12	4	18	68 15 24	Precentral_R
13	5	3	18 24 45	Frontal_Sup_R
14	5	4	35 15 51	Frontal_Mid_R
15	5	5	22 −2 57	Frontal_Sup_R
16	6	5	15 −17 69	Frontal_Sup_R
17	6	6	1 −29 84	Paracentral_Lobule_L
18	6	14	−13 −15 74	Paracentral_Lobule_L
19	6	16	1 −14 74	Paracentral_Lobule_L
20	7	4	53 0 49	Frontal_Mid_R
21	7	5	35 −16 52	Precentral_R
22	7	7	58 −11 43	Postcentral_R
23	7	8	36 −25 46	Postcentral_R
24	7	19	49 −18 48	Postcentral_R
25	8	5	32 −31 77	Postcentral_R
26	8	6	18 −47 78	Parietal_Sup_R
27	8	8	32 −42 51	Parietal_Inf_R
28	8	9	33 −56 72	Parietal_Sup_R
29	9	6	3 −43 58	Precuneus_L
30	9	9	15 −60 60	Parietal_Sup_R
31	9	21	−2 −66 67	Precuneus_L
32	10	8	48 −53 50	Angular_R
33	10	9	36 −62 51	Parietal_Sup_R
34	10	10	41 −56 30	Angular_R
35	10	11	42 −79 42	Occipital_Mid_R
36	10	20	31 −59 43	Angular_R
37	11	11	21 −81 36	Occipital_Sup_R
38	12	12	−29 12 54	Frontal_Mid_L
39	12	14	−24 −1 64	Frontal_Sup_L
40	13	12	−36 12 26	Frontal_Inf_Oper_L
41	13	13	−38 6 23	Frontal_Inf_Oper_L
42	13	22	−53 17 26	Frontal_Inf_Tri_L
43	14	12	−32 −3 43	Precentral_L
44	14	13	−48 −14 39	Parietal_Inf_L
45	14	14	−26 −16 51	Precentral_L
46	14	15	−42 −27 47	Parietal_Inf_L
47	14	23	−43 −11 50	Postcentral_L
48	15	6	−12 −43 78	Postcentral_L
49	15	14	−17 −28 62	Postcentral_L
50	15	15	−32 −42 58	Parietal_Sup_L

Table Footnotes: Channels, created by sources and detectors, were localized with Montreal Neurological Institute (MNI) coordinates and anatomical landmarks, as identified with the automated anatomical labeling (AAL) atlas. Short-separator channels are listed in blue font.

**Table 2.** HbO values for LE subtask.

*Dual vs. Single Motor*						
Channel	AAL Location	Dual task HbO mean (SD)	Single motor HbO mean (SD)	Adjusted *p* value	Subjects removed for control analysis	Corrected *p* value
1 (S1–D1)	Frontal_Sup_R	0.148 (0.271)	0.042 (0.227)	0.041	2	0.048
4 (S2–D2)	Frontal_Inf_Tri_R	0.432 (0.435)	0.261 (0.357)	0.009	0	N/A
38 (S12–D12)	Frontal_Mid_L	0.347 (0.377)	0.145 (0.365)	0.0004	0	N/A
40 (S13–D12)	Frontal_Inf_Oper_L	0.074 (0.422)	−0.038 (0.418)	0.035	1	0.054
41 (S13–D13)	Frontal_Inf_Oper_L	0.346 (0.434)	0.224 (0.410)	0.016	2	0.023
*Dual vs. Single Cognitive*						
Channel	AAL Location	Dual task HbO mean (SD)	Single cognitive HbO mean (SD)	Adjusted *p* value	Subjects removed for control analysis	Corrected *p* value

1 (S1–D1)	Frontal_Sup_R	0.148 (0.271)	0.030 (0.251)	0.018	2	0.017
2 (S1–D2)	Frontal_Mid_R	0.208 (0.478)	0.012 (0.396)	0.009	0	N/A
3 (S2–D1)	Frontal_Mid_R	0.164 (0.359)	0.025 (0.311)	0.005	2	0.003
4 (S2–D2)	Frontal_Inf_Tri_R	0.432 (0.435)	0.131 (0.354)	0.002	0	N/A
5 (S3–D2)	Frontal_Mid_R	0.060 (0.383)	−0.077 (0.335)	0.040	0	N/A
9 (S4–D2)	Frontal_Inf_Tri_R	0.449 (0.521)	0.118 (0.430)	0.00004	0	N/A
11 (S4–D7)	Precentral_R	0.429 (0.568)	0.175 (0.388)	0.003	0	N/A
41 (S13–D13)	Frontal_Inf_Oper_L	0.346 (0.434)	0.184 (0.327)	0.025	2	0.041

Table Footnotes: HbO, Oxygenated Hemoglobin; S, Source; D, Detector; SD, Standard Deviation. Adjusted *p*-value indicates Sidak adjusted value for multiple comparisons. Subjects Removed for Control Analysis indicates the number of participants with Scalp Coupling Index (SCI) values <0.75 who were removed from analysis to confirm that they were not unduly influencing results for each significant channel. Corrected *p*-value is the new *p*-value with those subjects removed.

**Table 3.** HbR values for LE subtask.

*Dual vs. Single Motor*						
Channel	AAL Location	Dual task HbR mean (SD)	Single motor HbR mean (SD)	Adjusted *p* value	Subjects removed for control analysis	Corrected *p* value
9 (S4–D2)	Frontal_Inf_Tri_R	−0.457 (0.463)	−0.230 (0.418)	0.001	0	N/A
11 (S4–D7)	Precentral_R	−0.487 (0.420)	−0.300 (0.459)	0.0003	0	N/A
40 (S13–D12)	Frontal_Inf_Oper_L	−0.344 (0.440)	−0.191 (0.405)	0.040	1	0.015
41 (S13–D13)	Frontal_Inf_Oper_L	−0.322 (0.489)	−0.110 (0.500)	0.011	2	0.030
43 (S14–D12)	Precentral_L	−0.204 (0.459)	−0.049 (0.376)	0.013	3	0.024
*Dual vs. Single Cognitive*						
Channel	AAL Location	Dual task HbR mean (SD)	Single cognitive HbR mean (SD)	Adjusted *p* value	Subjects removed for control analysis	Corrected *p* value

2 (S1–D2)	Frontal_Mid_R	−0.496 (0.547)	−0.295 (0.414)	0.004	0	N/A
4 (S2–D2)	Frontal_Inf_Tri_R	−0.287 (0.519)	−0.116 (0.481)	0.016	0	N/A
5 (S3–D2)	Frontal_Mid_R	−0.372 (0.490)	−0.199 (0.433)	0.010	0	N/A
7 (S3–D4)	Frontal_Inf_Tri_R	−0.360 (0.509)	−0.164 (0.501)	0.018	0	N/A
9 (S4–D2)	Frontal_Inf_Tri_R	−0.446 (0.457)	0.020 (0.370)	0.00001	0	N/A
11 (S4–D7)	Precentral_R	−0.487 (0.411)	−0.200 (0.390)	0.001	0	N/A
17 (S6–D6)	Paracentral_Lobule_L	−0.318 (0.426)	−0.057 (0.385)	0.001	1	0.001
18 (S6–D14)	Paracentral_Lobule_L	−0.199 (0.491)	−0.011 (0.498)	0.037	1	0.052
38 (S12–D12)	Frontal_Mid_L	0.327 (0.495)	0.231 (0.433)	0.037	0	N/A

Table Footnotes: HbR, DeOxygenated Hemoglobin; S, Source; D, Detector; SD, Standard Deviation. Adjusted *p* value indicates Sidak adjusted value for multiple comparisons. Subjects Removed for Control Analysis indicates the number of participants with Scalp Coupling Index (SCI) values <0.75 who were removed from analysis to confirm that they were not unduly influencing results for each significant channel. Corrected *p* value is the new *p* value with those subjects removed.

**Table 4.** HbO values for UE subtask.

*Dual vs. Single Motor*						
Channel	AAL Location	Dual task HbO mean (SD)	Single motor HbO mean (SD)	Adjusted *p* value	Subjects removed for control analysis	Corrected *p* value
3 (S2–D1)	Frontal_Mid_R	−0.173 (0.340)	−0.030 (0.429)	0.023	0	N/A
43 (S14–D12)	Precentral_L	0.341 (0.408)	0.214 (0.369)	0.028	10	0.383
*Dual vs. Single Cognitive*						
Channel	AAL Name	Dual task HbO mean (SD)	Single cognitive HbO mean (SD)	Adjusted *p* value	Subjects removed for control analysis	Corrected *p* value

4 (S2–D2)	Frontal_Inf_Tri_R	−0.139 (0.451)	−0.292 (0.411)	0.009	0	N/A
9 (S4–D2)	Frontal_Inf_Tri_R	0.173 (0.503)	−0.085 (0.355)	0.002	0	N/A
11 (S4–D7)	Precentral_R	0.284 (0.534)	0.075 (0.424)	0.020	0	N/A
22 (S7–D7)	Postcentral_R	0.222 (0.561)	0.077 (0.435)	0.037	2	0.104
41 (S13–D13)	Frontal_Inf_Oper_L	0.333 (0.446)	0.200 (0.388)	0.036	4	0.007
46 (S14–D15)	Parietal_Inf_L	0.291 (0.501)	0.165 (0.434)	0.022	4	0.065

Table Footnotes: HbO, Oxygenated Hemoglobin; S, Source; D, Detector; SD, Standard Deviation. Adjusted *p* value indicates Sidak adjusted value for multiple comparisons. Subjects Removed for Control Analysis indicates the number of participants with Scalp Coupling Index (SCI) values <0.75 who were removed from analysis to confirm that they were not unduly influencing results for each significant channel. Corrected *p*-value is the new *p*-value with those subjects removed.

**Table 5.** HbR values for UE subtask.

*Dual vs. Single Motor*						
Channel	AAL Name	Dual task HbR mean (SD)	Single motor HbR mean (SD)	Adjusted *p* value	Subjects removed for control analysis	Corrected *p* value
15 (S5–D5)	Frontal_Sup_R	−0.174 (0.702)	−0.054 (0.705)	0.012	5	0.011
23 (S7–D8)	Postcentral_R	−0.127 (0.430)	0.023 (0.420)	0.007	10	0.096
41 (S13–D13)	Frontal_Inf_Oper_L	−0.120 (0.512)	−0.037 (0.512)	0.030	4	0.029
*Dual vs. Single Cognitive*						
Channel	AAL Location	Dual task HbR mean (SD)	Single cognitive HbR mean (SD)	Adjusted *p* value	Subjects removed for control analysis	Corrected *p* value

4 (S2–D2)	Frontal_Inf_Tri_R	−0.248 (0.463)	−0.088 (0.442)	0.010	0	N/A
9 (S4–D2)	Frontal_Inf_Tri_R	−0.019 (0.501)	0.139 (0.437)	0.016	0	N/A
11 (S4–D7)	Precentral_R	−0.087 (0.485)	0.071 (0.473)	0.110	0	N/A
15 (S5–D5)	Frontal_Sup_R	−0.174 (0.702)	0.011 (0.550)	0.013	5	0.029
17 (S6–D6)	Paracentral_Lobule_L	0.015 (0.493)	0.197 (0.470)	0.023	2	0.034
39 (S12–D14)	Frontal_Sup_L	−0.050 (0.525)	0.151 (0.467)	0.001	4	0.001

Table Footnotes: HbR, DeOxygenated Hemoglobin; S, Source; D, Detector; SD, Standard Deviation. Adjusted *p* value indicates Sidak adjusted value for multiple comparisons. Subjects Removed for Control Analysis indicates the number of participants with Scalp Coupling Index (SCI) values <0.75 who were removed from analysis to confirm that they were not unduly influencing results for each significant channel. Corrected *p*-value is the new *p*-value with those subjects removed.

## Data Availability

Behavioral and neuroimaging datasets are available upon request. Please contact the corresponding author.
